# Sweetened beverage intake and risk of incident kidney stone: results from the UK Biobank

**DOI:** 10.3389/fnut.2026.1844118

**Published:** 2026-05-21

**Authors:** Binxian Ye, Yueming Liu, Jinshi Zhang, Jianguang Gong, Bin Zhu, Wangfang Chen

**Affiliations:** Urology & Nephrology Center, Department of Nephrology, Zhejiang Provincial People's Hospital, Affiliated People's Hospital, Hangzhou Medical College, Hangzhou, Zhejiang, China

**Keywords:** artificially sweetened beverages, genetic risk, KSD, natural juices, sugary beverage

## Abstract

**Background:**

To investigate the associations between consumption of sugar-sweetened beverages (SSBs), artificially sweetened beverages (ASBs), and natural juices (NJs) and incident kidney stone disease (KSD), and to assess whether genetic susceptibility modifies these associations.

**Methods:**

We conducted a prospective cohort study of 191,863 UK Biobank participants free of KSD at baseline. Beverage consumption was assessed using web-based 24-h dietary recalls (2009–2012), categorized as 0, 0.1–1.0, 1.1–2.0, and >2.0 units/day (1 unit = 250 ml). A polygenic risk score (PRS) comprising 22 single nucleotide polymorphisms quantified genetic susceptibility. Multivariable Cox regression models estimated hazard ratios (HRs) and 95% confidence intervals (CIs), with gene-environment interactions evaluated through stratified analyses.

**Results:**

During 13.5 years mean follow-up, 2,024 incident KSD cases occurred. Compared with non-consumers, participants consuming >2 units/day of SSBs had 51% higher KSD risk (HR 1.51, 95% CI 1.17–1.95) with significant dose-response relationship (*P* for trend < 0.001). ASBs showed elevated risk at 1.1–2.0 units/day (HR 1.25, 95% CI 1.02–1.28) without linear trend (*P* = 0.192). NJs showed no significant association (*P* for trend = 0.419). Higher PRS independently predicted KSD risk but did not significantly modify beverage-KSD associations (all P for interaction >0.05). Joint analyses revealed highest KSD hazard among participants with high SSB intake and low PRS (HR 2.09, 95% CI 1.46–2.98).

**Conclusion:**

Higher SSB consumption is dose-dependently associated with increased KSD risk, while ASBs show non-linear associations and NJs demonstrate no significant association. Genetic susceptibility independently predicts KSD risk but does not substantially modify beverage-KSD relationships. Limiting SSB intake represents an important modifiable strategy for KSD prevention independent of genetic predisposition.

## Introduction

Kidney stone disease (KSD), defined by the formation of calculi in the kidneys, ureter, bladder, or urethra, imposes a substantial global burden of morbidity, disability, and health-care costs (1). Global incidence of KSD has decreased in recent years, while the mortality rate has plateaued worldwide, suggesting improvements in risk factors but persistently limited access to and quality of urological health care, as well as marked heterogeneity across regions and countries (2). Furthermore, KSD has been implicated as a risk factor for several systemic disorders, notably kidney failure (3) and coronary heart disease (4).

The etiology of KSD is multifactorial, involving genetic variation, dietary factors, and underlying metabolic disorders (5). For individuals with a prior episode of KSD, augmentation of fluid intake is routinely recommended. Evidence from multiple studies (6, 7) has demonstrated the critical role of fluid intake in modulating the risk of incident kidney stone formation. However, the type of fluid intake may also influence stone risk. For example, consumption of sodas (8) has been associated with a higher likelihood of stone formation, while beverages such as coffee and tea (8, 9) have been reported to confer a protective effect.

Sugary beverages (SBs) are classified into three major categories: sugar-sweetened beverages (SSBs), artificially sweetened beverages (ASBs), and natural juices (NJs). SSBs, including flavored lattes, sodas, fruit-flavored drinks, and energy drinks, are consumed on a global scale, with intake in many high-income countries exceeding the recommended daily limits for free sugar and continuing to rise in low- and middle-income countries (10). Regular consumption of SSBs has been associated with increased mortality risk (11), a higher likelihood of developing multimorbidity, and an increased number of chronic conditions (12). Two potential alternatives are ASBs (i.e., zero- or low-calorie beverages) and NJs (100% pure fruit or vegetable juices), which, compared to SSBs, may be associated with a lower risk of cardiometabolic diseases (13, 14). Although the relationship between different types of SBs and the risk of KSD is of considerable clinical significance, robust evidence from high-quality, large-scale prospective studies is still lacking. A cross-sectional analysis of 15,779 young and middle-aged adults from the National Health and Nutrition Examination Survey suggested (15) that higher sugar-sweetened beverage intake is associated with an increased risk of kidney stones, although specific beverage types were not further investigated.

Genetic factors, together with lifestyle factors, also play a significant role in increasing the risk of kidney stone formation. Previous genome-wide association studies (16, 17) have identified multiple loci associated with KSD. However, the interaction between genetic risk factors and SBs in relation to KSD risk remains poorly understood. To date, no study has systematically evaluated whether genetic susceptibility modifies the association between SBs intake and the incidence of KSD.

Against this background, we examined the long-term associations of SSBs, ASBs and NJs with incident KSD in the UK Biobank, a large population-based cohort in the United Kingdom. Specifically, we (i) assessed whether SSB, ASB and NJ intake is associated with a higher risk of KSD, and (ii) constructed a KSD-specific PRS to evaluate whether genetic susceptibility modifies the SB–KSD association.

## Methods

### Study design and population

We utilized data from the UK Biobank, a population-based prospective cohort study that enrolled over 500,000 community-dwelling adults, aged 37–73 years, from across the United Kingdom between 2006 and 2010. The study's design, recruitment approach, and detailed protocols have been thoroughly outlined in previous publications (18).

**Figure 1:**
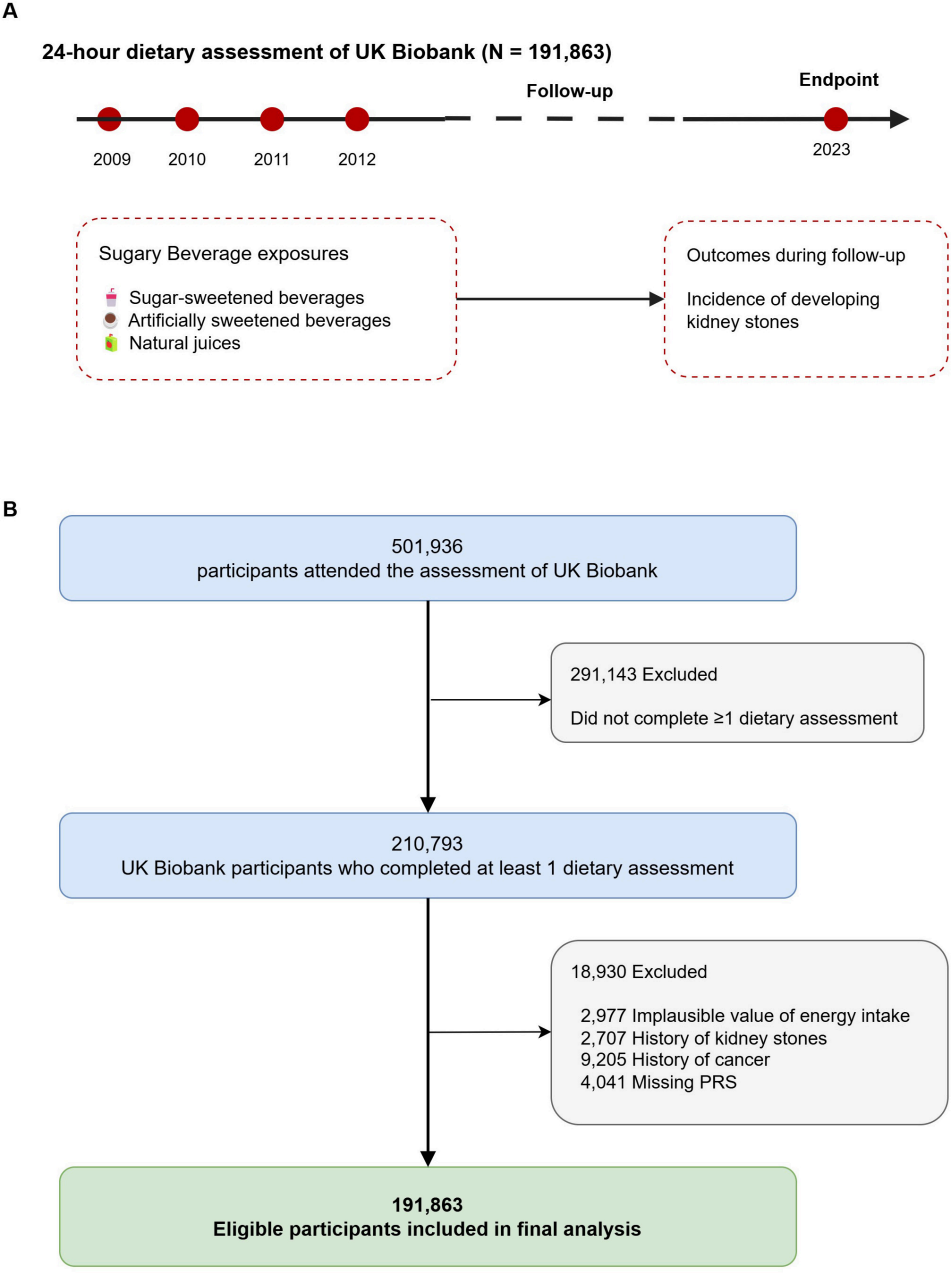
Study design and selection process diagram. **(A)** Conceptual framework of the study design. **(B)** Flowchart of the selection process.

Participants who completed at least one online dietary questionnaire in the UK Biobank were included in the analysis, resulting in a total of 210,793 individuals. Exclusion criteria included: (1) non-typical dietary patterns or implausible energy intake (< 500 or >3,500 kcal/day for women; < 800 or >4,000 kcal/day for men; *n* = 2,977); (2) prevalent KSD at enrollment (*n* = 2,707); (3) prior diagnosis of malignant neoplasm (*n* = 9,205); and (4) unavailable genetic data (*n* = 4,021). Following these exclusions, 191,863 participants were included in the final analysis (Figure 1).

### Assessment of SBs consumption

Beverage consumption was evaluated using web-based 24-h dietary recall questionnaires, which were completed by participants every 3–4 months from 2009 to 2012. At each time point, participants documented the quantity of beverages consumed (measured in units: glasses/cans/cartons, with one unit equivalent to 250 mL) during the preceding 24-h period. Consistent with prior research (11), daily beverage intake was stratified into four categories: 0, 0.1–1.0, 1.1–2.0, and >2.0 units per day. SBs were categorized as: (1) SSBs, comprising fizzy drinks and squash; (2) ASBs, including low-calorie/diet drinks; and (3) NJs, encompassing pure orange juice, grapefruit juice, and other 100% fruit or vegetable juices. For individuals with multiple dietary assessments, the average beverage intake was computed across all completed recalls.

### Ascertainment of KSD

This study focused on incident KSD, defined as newly diagnosed cases during follow-up. Case ascertainment was performed using multiple linked data sources: (1) ICD-10 diagnostic codes extracted from hospital inpatient episodes and national death registers; (2) OPCS-4 procedural codes obtained from hospital admission records; and (3) self-reported surgical intervention codes specific to KSD treatment (Supplementary Table S1).

### Polygenic risk score for KSD

Genetic data processing, quality control, and imputation procedures in the UK Biobank have been extensively documented elsewhere (19). In this study, we calculated a polygenic risk score (PRS) to quantify each participant's burden of common genetic variants associated with KSD risk, following the strategy reported in an earlier study (20). To quantify inherited susceptibility to KSD, we constructed a PRS based on 22 independent single nucleotide polymorphisms (SNPs) with validated associations with KSD risk (Supplementary Table S2). Each SNP was encoded according to risk allele dosage (0, 1, or 2 copies), and the composite PRS was calculated as a weighted sum of risk alleles, with weights corresponding to published effect estimates (β-coefficients) to reflect the relative contribution of each variant to overall genetic liability. To assess the predictive performance of PRS for KSD, we calculated Harrell's C-index based on a Cox proportional hazards model including the PRS, age, sex, and the first 10 principal components of ancestry.

### Assessment of covariates

To control for potential confounding, we collected baseline data on demographic characteristics, lifestyle factors, and clinical measurements. Demographic variables included age, sex, ethnicity, educational attainment, Townsend deprivation index, and household income. Lifestyle factors comprised physical activity level, body mass index (BMI), smoking status, alcohol consumption, and daily water intake. Clinical parameters included estimated glomerular filtration rate (eGFR) and a composite health score. Dietary quality was assessed using a total diet score (range: 0–7), calculated by assigning one point for each dietary component meeting recommended intake guidelines, with higher scores indicating healthier dietary patterns. Detailed scoring methodology has been described previously (21).

### Statistical analysis

Baseline characteristics of participants are presented stratified by SB intake categories. Normally distributed continuous variables are expressed as mean ± standard deviation (SD), while non-normally distributed variables are reported as median [interquartile range (IQR)]. Categorical variables are summarized as frequencies and percentages. Missing covariate data were addressed using multiple imputation strategies, including single imputation, missing indicator methods, and multiple imputation by chained equations (details in Supplementary Table S3). Cox proportional hazards regression models were used to estimate the association between SB intake and incident KSD, with results presented as hazard ratios (HRs) and 95% confidence intervals (CIs). Person-time was calculated from baseline until the earliest of KSD diagnosis, death, loss to follow-up, or administrative censoring at study end. Three sequential models were constructed: Model 1 was unadjusted; Model 2 adjusted for age and sex; and Model 3 (fully adjusted) additionally included BMI, ethnicity, educational attainment, alcohol consumption, Townsend deprivation index, household income, smoking status, physical activity level, healthy diet score, and eGFR. To evaluate the robustness of our findings to potential unmeasured confounding, we calculated E-values (22) for the point estimates and the lower bounds of the 95% confidence intervals for all primary associations. Because the outcome was rare at the end of follow-up (< 15%), HRs were treated as approximations to RRs and were not additionally converted before E-value calculation. The E-value was calculated as $E-value=HR+\;\sqrt{HR\left(HR-1\right)}$ for HRs ≥1. For confidence intervals, the E-value was based on the confidence-limit closest to the null, with an interval E-value of 1 when the confidence interval crossed the null. To explore potential non-linear associations, we fitted restricted cubic splines (RCS) with four knots at pre-specified percentiles in fully adjusted models. For participants with SB intake coded as 600, the value was recoded to 6 units/day. Similar Cox regression and RCS analyses were performed to assess the association between PRS and KSD incidence, characterizing dose–response relationships across the genetic risk spectrum.

To examine whether genetic susceptibility modified the association between SB intake and incident KSD, we stratified participants into low- and high-genetic-risk groups based on the median PRS value. We then constructed Cox proportional hazards models incorporating multiplicative interaction terms between PRS strata (low vs. high) and SB exposure variables (SSB, ASB, or NJ intake, modeled separately). Participants with zero SB intake and low genetic risk constituted the joint reference category. Statistical significance of gene-environment interactions was evaluated using likelihood ratio tests (LRTs), comparing nested models with and without the PRS × intake interaction term.

To explore potential effect modification, we conducted subgroup analyses stratified by age, sex, daily water intake, eGFR, and BMI. Multiplicative interactions between each stratifying variable and SB intake were formally tested by adding cross-product terms to the fully adjusted model (Model 3), with *P* values for interaction derived from likelihood ratio tests. To assess the robustness of our findings, we performed several sensitivity analyses: (1) complete-case analysis, restricting the sample to participants with no missing covariate data; (2) limiting the cohort to individuals with at least two dietary assessments to improve exposure measurement accuracy; (3) mutually adjusting for all three SB types (SSB, ASB, NJ) simultaneously in multivariable models to estimate independent associations of each beverage category with incident KSD; and (4) conducting a sensitivity analysis using the Fine-Gray competing risks model to adjust for competing bias from mortality.

All data management and statistical analyses were conducted using R via Posit Workbench (formerly RStudio Server, version 2.2.1) on the UK Biobank Research Analysis Platform (RAP). All statistical tests were two-sided, with *P* < 0.05 considered statistically significant unless otherwise specified.

## Results

### Baseline characteristics of participants

Baseline characteristics of the study population, stratified by beverage consumption, are presented in Table 1 and Supplementary Table S4. Among 191,863 participants free of KSD at baseline, the mean (SD) age was 55.9 (7.9) years, and 55.2% were female. During a mean follow-up of 13.5 years [median (IQR): 13.7 (13.1–14.5) years], 2,024 incident KSD cases were identified. Compared with non-consumers or low consumers, participants with higher SSB intake were more likely to be younger, male, have higher BMI, be current smokers, and reside in more socioeconomically deprived areas (higher Townsend Deprivation Index). Detailed baseline characteristics stratified by ASB and NJ consumption are provided in Supplementary Table S4.

**Table 1:** Baseline characteristics of participants according to sugar-sweetened beverage intake (*n* = 191,863).

Categories	Overall	Intake categories of sugar-sweetened beverage			
		None	>0–1 units/d	>1–2 units/d	>2 units/d
N	191,863	129,675	49,635	9,013	3,540
Age [mean (sd)]	55.92 (7.94)	56.30 (7.83)	55.58 (8.03)	53.73 (8.19)	52.49 (8.12)
BMI [mean (sd)]^a^	26.91 (4.62)	26.78 (4.58)	27.01 (4.57)	27.61 (4.91)	28.35 (5.39)
Underweight	1,030 (0.5)	754 (0.6)	231 (0.5)	33 (0.4)	12 (0.3)
Normal	71,060 (37.0)	49,384 (38.1)	17,819 (35.9)	2,871 (31.9)	986 (27.9)
Overweight	80,007 (41.7)	53,722 (41.4)	21,008 (42.3)	3,809 (42.3)	1,468 (41.5)
Obese	39,766 (20.7)	25,815 (19.9)	10,577 (21.3)	2,300 (25.5)	1,074 (30.3)
Gender (%)					
Female	105,939 (55.2)	73,761 (56.9)	26,512 (53.4)	4,195 (46.5)	1,471 (41.6)
Male	85,924 (44.8)	55,914 (43.1)	23,123 (46.6)	4,818 (53.5)	2,069 (58.4)
Drinking status (%)					
Never	6,077 (3.2)	3,725 (2.9)	1,770 (3.6)	383 (4.2)	199 (5.6)
Previous	5,737 (3.0)	3,549 (2.7)	1,574 (3.2)	405 (4.5)	209 (5.9)
Current	180,049 (93.8)	122,401 (94.4)	46,291 (93.3)	8,225 (91.3)	3,132 (88.5)
Ethnicity (%)					
Non_white	8,004 (4.2)	4,892 (3.8)	2,324 (4.7)	552 (6.1)	236 (6.7)
White	183,859 (95.8)	124,783 (96.2)	47,311 (95.3)	8,461 (93.9)	3,304 (93.3)
TDI [mean (sd)]	−1.59 (2.87)	−1.61 (2.85)	−1.63 (2.85)	−1.34 (3.00)	−1.10 (3.11)
Smoking status (%)					
Never	109,318 (57.0)	72,806 (56.1)	29,309 (59.0)	5,204 (57.7)	1,999 (56.5)
Previous	67,541 (35.2)	46,728 (36.0)	16,725 (33.7)	2,948 (32.7)	1,140 (32.2)
Current	15,004 (7.8)	10,141 (7.8)	3,601 (7.3)	861 (9.6)	401 (11.3)
Education (%)					
College	82,180 (42.8)	56,317 (43.4)	21,265 (42.8)	3,410 (37.8)	1,188 (33.6)
Other levels	92,787 (48.4)	61,592 (47.5)	24,365 (49.1)	4,820 (53.5)	2,010 (56.8)
Unknown	16,896 (8.8)	11,766 (9.1)	4,005 (8.1)	783 (8.7)	342 (9.7)
Household income, €/y (%)					
Less than 18,000	29,310 (15.3)	19,823 (15.3)	7,351 (14.8)	1,480 (16.4)	656 (18.5)
18,000 to 30,999	46,207 (24.1)	31,266 (24.1)	11,954 (24.1)	2,147 (23.8)	840 (23.7)
31,000 to 51,999	54,768 (28.5)	36,714 (28.3)	14,431 (29.1)	2,602 (28.9)	1,021 (28.8)
52,000 to 100,000	47,484 (24.7)	32,075 (24.7)	12,416 (25.0)	2,171 (24.1)	822 (23.2)
Greater than 100,000	14,094 (7.3)	9,797 (7.6)	3,483 (7.0)	613 (6.8)	201 (5.7)
Physical activity (%)					
Non	72,783 (37.9)	48,780 (37.6)	19,258 (38.8)	3,429 (38.0)	1,316 (37.2)
Yes	119,080 (62.1)	80,895 (62.4)	30,377 (61.2)	5,584 (62.0)	2,224 (62.8)
Score diet [mean (sd)]	2.87 (1.28)	2.95 (1.27)	2.76 (1.27)	2.61 (1.30)	2.51 (1.31)
eGFR [mean (sd)]	95.82 (12.53)	95.80 (12.36)	95.60 (12.79)	96.65 (13.04)	97.26 (13.40)
Drinking water (%)					
< 3	92,394 (48.2)	61,541 (47.5)	25,106 (50.6)	4,266 (47.3)	1,481 (41.8)
> = 3	84,530 (44.1)	58,155 (44.8)	20,431 (41.2)	4,118 (45.7)	1,826 (51.6)
Unknown	14,939 (7.8)	9,979 (7.7)	4,098 (8.3)	629 (7.0)	233 (6.6)

BMI, body mass index; eGFR, estimated glomerular filtration rate; LDL-C, low-density lipoprotein cholesterol; TDI, Townsend Deprivation Index.

^a^Body weight status was defined by BMI ( ≤ 20 kg/m2 to be underweight, >20 and ≤ 25 to be normal weight, >25 and ≤ 30 to be overweight, and >30 to be obesity).

### Association of the consumption of 3 types of beverages with incident KSD

**Figure 2:**
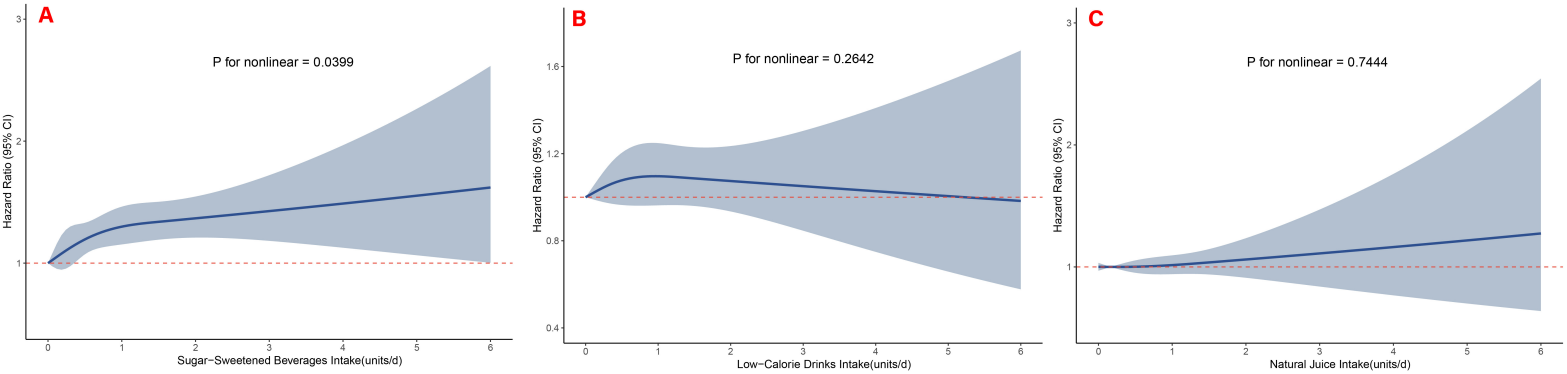
Restricted cubic spline models for the association between beverage intake categories and the incidence of kidney stone development during follow-up. **(A)** Relationship between sugar-sweetened beverage intake and KSD; **(B)** Relationship between low-calorie drink intake and KSD; **(C)** Relationship between natural juice intake and KSD.

All three SB types were associated with incident KSD risk (Table 2). In fully adjusted Cox proportional hazards models (Model 3), compared with non-consumers, participants consuming >2 units/day of SSBs had a 51% higher risk of KSD (HR 1.51, 95% CI 1.17–1.95). A significant dose-response relationship was observed between SSB consumption and KSD incidence (*P* for trend < 0.001). For ASB consumption, the adjusted HRs were 1.05 (95% CI 0.93–1.18) for >0–1 unit/day, 1.25 (95% CI 1.02–1.28) for >1–2 units/day, and 0.94 (95% CI 0.69–1.28) for >2 units/day, compared with non-consumers. Although an elevated risk was observed in the intermediate consumption category, no significant linear trend was detected (*P* for trend = 0.192), suggesting a potential non-linear association. In contrast, NJ consumption showed no significant association with KSD risk across all consumption categories (*P* for trend = 0.419). Consistent associations were observed between the consumption of all three categories of SBs and the risk of KSD (Figure 2). For SSBs, stratified analyses suggested that the associations were more pronounced among females, participants aged ≥60 years, those with eGFR ≥98.1 ml/min/1.73 m^2^, and individuals with BMI < 25.0 kg/m^2^. However, with the exception of eGFR, no statistically significant interactions were observed (all P for interaction >0.05). For ASB and NJ, subgroup analyses showed trends generally consistent with the primary analyses (Supplementary Table S5). Sensitivity analyses yielded results consistent with the primary findings. The associations remained robust when restricted to participants with complete covariate data (Supplementary Table S6), those who completed ≥2 dietary assessments (Supplementary Table S7), when the three SB types were mutually adjusted in the same model (Supplementary Table S8) and when conducted the Fine-Gray competing risks model to adjust for competing bias from mortality (Supplementary Table S9).

**Table 2:** Risk of incident kidney stone by category of beverage intake.

Categories	Cases/total	Person-years	Model1	Model2	Model3	*E*-value (CI)
Sugar-sweetened beverage (units/d)						
0, HR (95% CI)	1,236/129,675	1,756,946	1 (reference)	1 (reference)	1 (reference)	
>0–1, HR (95% CI)	578/49,635	672,393	1.22(1.11, 1.35)	1.20(1.09, 1.33)	1.18(1.07, 1.30)	1.64(1.34)
>1–2, HR (95% CI)	147/9,013	121,448	1.72 (1.45, 2.04)	1.66 (1.40, 1.97)	1.51 (1.27, 1.79)	2.39 (1.86)
>2, HR (95% CI)	63/3,540	47,198	1.90 (1.47, 2.45)	1.80 (1.40, 2.32)	1.51 (1.17, 1.95)	2.39 (1.62)
*P*-trend			< 0.001	< 0.001	< 0.001	
Artificially-sweetened beverage (units/d)						
0, HR (95% CI)	1,552/152,166	2,060,048	1 (reference)	1 (reference)	1 (reference)	
>0–1, HR (95% CI)	330/29,271	397,081	1.10 (0.98, 1.24)	1.16 (1.03, 1.31)	1.05 (0.93, 1.18)	1.28 (1.00)
>1–2, HR (95% CI)	100/6,915	93341	1.42 (1.16, 1.74)	1.53 (1.25, 1.88)	1.26 (1.03, 1.55)	1.83 (1.21)
>2, HR (95% CI)	42/3,511	47,515	1.17 (0.86, 1.59)	1.29 (0.95, 1.75)	0.94 (0.69, 1.28)	1.32 (1.00)
*P*-trend			0.001	< 0.001	0.192	
Naturally sweet juices (units/d)						
0, HR (95% CI)	997/92,823	1,252,821	1 (reference)	1 (reference)	1 (reference)	
>0–1, HR (95% CI)	875/85,251	1,158,284	0.95 (0.87, 1.04)	0.91 (0.84, 1.00)	1.01 (0.99, 1.11)	1.00 (1.00)
>1–2, HR (95% CI)	123/11,852	160,837	0.96 (0.80, 1.16)	0.90 (0.75, 1.09)	1.01 (0.99, 1.22)	1.00 (1.00)
>2, HR (95% CI)	29/1,931	26,044	1.40 (0.97, 2.02)	1.27 (0.88, 1.84)	1.34 (0.93, 1.94)	2.01 (1.00)
*P*-trend			0.843	0.234	0.419	

HR, hazard ratio; NA, not applicable. Model 1: unadjusted; Model 2: adjusted for age and gender; Model 3: model 2 also adjusted for BMI, ethnicity, education, drinking status, Townsend deprivation index, household income, smoking status, physical activity, healthy diet score, and eGFR.

### Joint effect of SBs and genetic susceptibility

**Figure 3:**
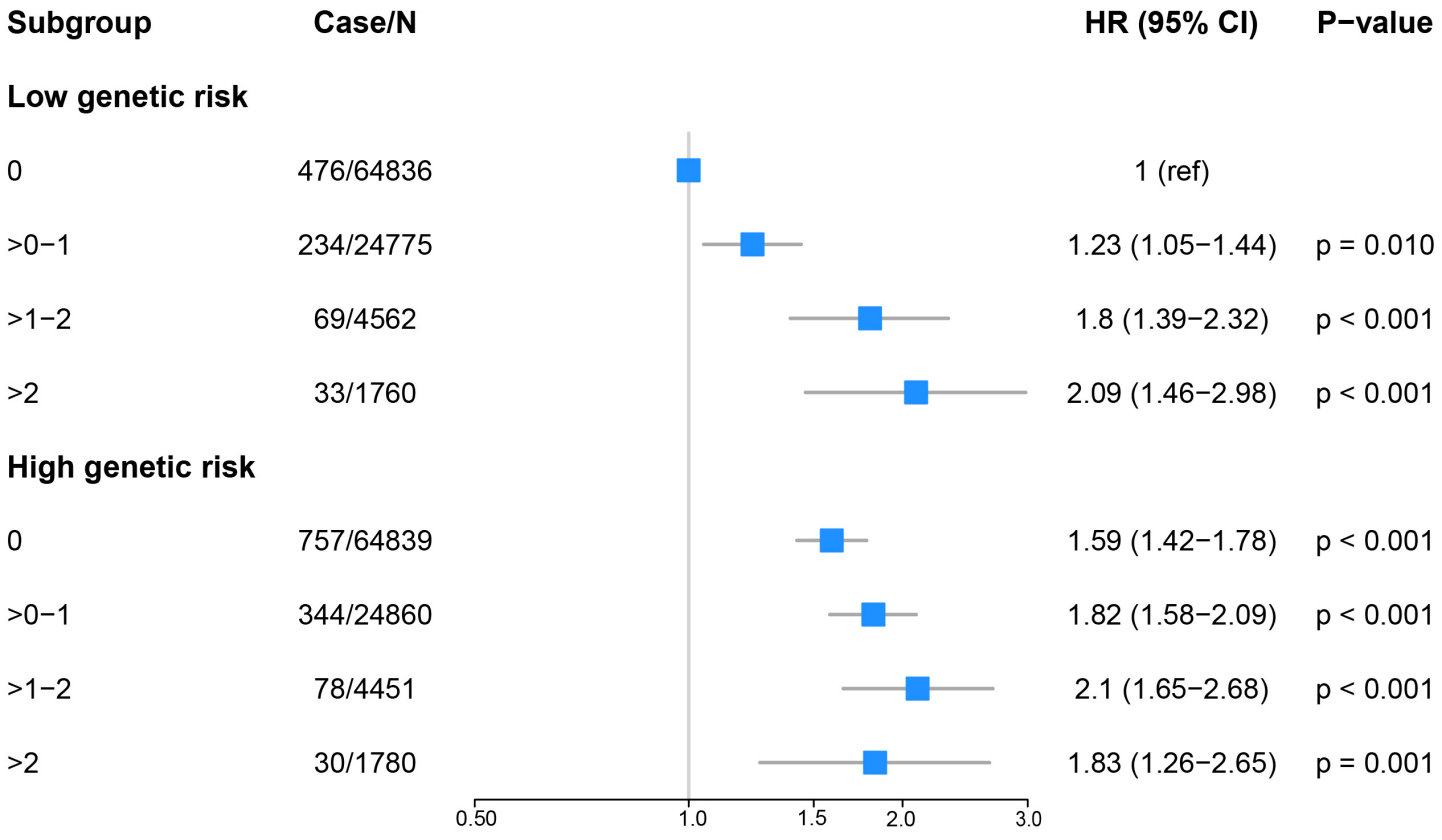
Joint effects of polygenic risk score (PRS) with sugar-sweetened beverage intake on kidney stone risk (*n* = 191,863). *P*-interactions = 0.050 for SSB × PRS. The blue boxes indicate the HRs, and the gray whiskers represent the CIs. HRs were calculated from Cox proportional hazard models adjusted for age, gender, BMI, ethnicity, education, drinking status, Townsend deprivation index, household income, smoking status, physical activity, healthy diet score and eGFR.

We observed that higher PRS was significantly and positively associated with increased KSD risk in a dose-dependent manner (Supplementary Figure S1). The predictive performance of the PRS for KSD was evaluated using Harrell's C-index. In the Cox proportional hazards model adjusted for age, sex, and the first 10 principal components of ancestry, the C-index was 0.636 (95% CI: 0.624–0.648), indicating modest discrimination. These associations were not materially modified by PRS (P for interaction = 0.050 for SSB × PRS, 0.133 for ASB × PRS, and 0.537 for NJ × PRS; Figure 3 and Supplementary Figures S2, S3). In joint association analyses, the highest KSD hazard was observed among participants with high SSB intake (>2 units/day) and low PRS (HR 2.09; 95% CI 1.46, 2.98), as well as those with moderate-to-high SSB intake (>1–2 units/day) and low PRS (HR 2.10; 95% CI 1.65, 2.68), compared with participants reporting no SSB intake and low PRS (Figure 3). Similarly, for ASB, the highest KSD hazard was observed among participants with moderate-to-high intake (>1–2 units/day) and low PRS (HR 1.90; 95% CI 1.46, 2.47), relative to those with no ASB intake and low PRS (Supplementary Figure S2). For NJ, the highest hazard was observed among participants with high intake (>2 units/day) and low PRS (HR 1.75; 95% CI 1.03, 2.98), compared with those reporting no NJ intake and low PRS (Supplementary Figure S3).

## Discussion

This study leveraged data from the large-scale UK Biobank cohort to investigate the associations between consumption of three types of SBs and the risk of incident KSD. We found that higher intake of SSBs was associated with an increased risk of KSD in a dose-dependent manner, whereas ASB intake was associated with elevated risk primarily at moderate-to-high levels (1.0–2.0 units/day), and NJ intake showed no significant association with KSD risk. These associations were generally consistent across major subgroups and were not materially modified by genetic susceptibility to KSD. To our knowledge, this is the largest prospective cohort study to date examining the potential impact of sugary beverage consumption on KSD risk and the first to explore both the potential interaction and joint effects of sugary beverage intake and genetic predisposition on incident KSD.

Previous studies (8, 15, 23–25) have reported inconsistent findings regarding the associations between consumption of different beverage types and KSD risk. A prospective study encompassing three ongoing cohorts found that (8) participants in the highest category of sugar-sweetened cola consumption had a 23% higher risk of developing kidney stones compared with those in the lowest category (HR 1.23; 95% CI 1.04, 1.45). Similarly, sugar-sweetened noncola beverages were associated with a 33% higher risk (HR 1.33; 95% CI 1.12, 1.58), while artificially sweetened noncola beverages showed a marginally significant elevated risk. Consistent with these findings, a cross-sectional analysis of 15,779 nationally representative participants from the National Health and Nutrition Examination Survey (NHANES) demonstrated that (15) both absolute SSB intake (per 100 kcal/d) and relative intake (per 1% of total energy) were positively associated with kidney stone risk, with odds ratios (ORs) of 1.065 (95% CI 1.038, 1.093) and 1.015 (95% CI 1.009, 1.022), respectively. However, the Health Professionals Follow-up Study, which included 51,529 male participants over 6 years of follow-up, did not observe (24) any significant association between high consumption of SSBs and KSD development. NJs are often consumed as a perceived healthier alternative to SSBs due to their natural origin and nutrient content. However, the evidence regarding their association with kidney stone risk remains equivocal, with some studies suggesting a protective effect (26, 27), while others have reported null findings (8, 24). The inconsistency in previous findings may stem from substantial heterogeneity across studies, encompassing variations in cohort characteristics (e.g., age, sex, and ethnicity), study design (prospective vs. cross-sectional), sample sizes, dietary assessment methodologies (e.g., food frequency questionnaires vs. 24-h dietary recalls), follow-up duration, and outcome definitions for KSD (e.g., self-reported vs. clinically confirmed). In contrast to these inconsistent prior findings, our study revealed a dose-dependent positive association between SSB intake and KSD risk, whereas ASB consumption demonstrated a marginally significant association only at higher intake levels. These observations highlight the necessity of accounting for the potential detrimental effects of both SSBs and ASBs on KSD development. By addressing key methodological limitations of prior research, our findings strengthen the evidence base supporting dietary recommendations to restrict SSB and ASB consumption as a preventive strategy for KSD.

Kidney stone formers tend to retain and accumulate crystals within the renal parenchyma, ultimately leading to stone formation. Although the biological mechanisms underlying the association between SBs and KSD are not fully elucidated, several plausible pathways may explain our findings. SSBs, recognized as a major dietary source of free sugars, are characterized by a high glycemic load (28), and elevated sugar intake has been consistently associated with an increased urinary calcium-to-creatinine ratio (29), a well-established marker of heightened stone formation risk. Moreover, SSB consumption is linked to elevated serum uric acid levels and increased renin secretion, which may directly or indirectly contribute to renal interstitial fibrosis and subsequent stone development (30). Beyond these immediate urinary effects, habitual SSB consumption promotes weight gain and insulin resistance, thereby exacerbating uric acid stone risk while potentially amplifying calcium stone formation (31). A behavioral “substitution” pathway also warrants consideration (32), as SSBs may displace water intake, resulting in reduced urine volume and increased concentration of lithogenic solutes. Low urine volume represents a broadly validated risk factor across all stone types. ASBs have emerged as popular substitutes for SSBs (33), especially among those seeking to mitigate metabolic syndrome and obesity-associated health risks. Nevertheless, our results, in line with previous research (8), suggest that ASBs confer no protective advantage against KSD; on the contrary, ASB consumption demonstrated a borderline significant elevation in KSD risk. One plausible mechanism is ASB intake has been implicated in promoting adiposity and body weight increase irrespective of caloric consumption, possibly mediated by modifications in intestinal microbiome profiles and glycemic regulation (34). In this study, natural juice consumption showed no significant association with KSD risk. The relationship between juice intake and nephrolithiasis is multifaceted, likely contingent upon juice type. Citrus-based juices, notably orange and lemon juice, have been extensively studied for their lithogenic protective properties (8, 26), as citric acid serves as a potent inhibitor of calcium oxalate crystallization by complexing with urinary calcium and elevating urinary pH. Conversely, high-fructose juices such as apple and grape juice (26) may paradoxically augment stone risk through enhanced urinary calcium and uric acid excretion, promoting crystal nucleation. This dichotomy highlights the necessity of considering juice composition when assessing nephrolithiasis risk.

Twin studies have demonstrated that KSD exhibits a heritability of approximately 56% (9), underscoring the substantial genetic contribution to disease susceptibility. Accumulating evidence further highlights the interplay of genetic, environmental, and lifestyle factors in KSD pathogenesis (35). Consistent with these observations, our study revealed that the PRS served as a robust independent predictor of incident KSD in a dose-dependent manner, reinforcing the clinical utility of genetic risk stratification. Although no statistically significant interaction was detected between overall SB consumption and genetic risk, SSB intake exhibited a borderline interaction, suggesting that curtailing SSB consumption may be particularly critical for individuals with heightened genetic susceptibility. This finding, together with the observation of a high hazard ratio (HR) in the high and moderate-to-high SSB intake groups among individuals with a low polygenic risk score (PRS), calls for further explanation. Based on our results and the existing literature, we propose the following potential mechanisms: Although a high HR was observed in the low-PRS group with high SSB intake, this finding suggests that the adverse effect of SSB may operate, at least in part, independently of known genetic risk loci. Potential mechanisms include SSB-induced metabolic disturbances (e.g., high glycemic load, uric acid elevation, hepatic fat accumulation) that directly promote disease development, and these effects may be particularly pronounced in individuals who lack strong genetic protection. Furthermore, a low PRS indicates a low burden of currently known risk alleles but does not signify the complete absence of genetic susceptibility. Future studies incorporating more comprehensive genomic data (e.g., genome-wide PRS, epigenetic marks) and cumulative measures of SSB exposure are needed to further elucidate the underlying biology. With respect to ASBs, associations appeared more pronounced among those with elevated genetic risk: even at approximately 2 units/day, ASB consumption retained a positive, dose-dependent association with KSD incidence in this subgroup. While these findings necessitate cautious interpretation given the limited statistical power for interaction testing, they provide preliminary evidence that genetic susceptibility may modulate beverage–KSD associations. These observations align with the emerging paradigm of precision nutrition, wherein dietary recommendations are tailored to individual genetic profiles to optimize preventive strategies.

This study has several notable strengths. First, it leveraged the large-scale prospective design of the UK Biobank, encompassing over 190,000 participants with comprehensive phenotypic and lifestyle data, enabling rigorous adjustment for potential confounders. Second, multiple sensitivity and subgroup analyses consistently yielded robust associations, reinforcing the reliability and generalizability of our findings. Third, the availability of genome-wide genetic data facilitated construction of polygenic risk scores and in-depth exploration of gene-environment interactions, providing novel insights into how genetic susceptibility modulates the associations between beverage consumption and KSD pathogenesis.

Several limitations should be acknowledged. First, the observational design precludes causal inference, and residual confounding from unmeasured factors (e.g., overall diet quality, hydration adequacy, discretionary salt use) may persist despite comprehensive covariate adjustment. Second, SSB intake was assessed via self-reported 24-h dietary recalls, which are prone to measurement error and recall bias and may not capture habitual consumption or temporal changes in intake patterns. Although sensitivity analyses restricted to participants with repeated dietary assessments yielded consistent findings, we were unable to fully characterize long-term dietary trajectories or account for potential regression dilution bias. Third, kidney stone outcomes were ascertained through linkage to electronic health records, which may under ascertain cases managed in primary care settings and are subject to diagnostic misclassification, potentially attenuating the observed associations. Fourth, UK Biobank participants are generally healthier and of higher socioeconomic status than the general UK population, which may limit generalizability to more socioeconomically diverse populations and other geographic or ethnic groups. Fifth, our polygenic risk score captured only a modest fraction of genetic heritability, and statistical power for gene-environment interaction testing remained limited by the relatively low incidence rate despite the large sample size. Additionally, the predominantly European ancestry of participants restricts generalizability to non-European populations.

In conclusion, in this large prospective cohort study of 191,863 UK Biobank participants followed for a mean of 13.5 years, higher consumption of SSBs was associated with increased risk of incident KSD in a dose-dependent manner, with participants consuming >2 units/day demonstrating a 51% elevated risk compared with non-consumers. ASB consumption showed an elevated risk at intermediate intake levels (1.0–2.0 units/day), though without a clear linear dose-response relationship, while NJ consumption showed no significant association with KSD risk. Genetic susceptibility, assessed through a polygenic risk score, independently predicted KSD incidence but did not significantly modify the associations between sweetened beverage consumption and KSD risk, suggesting that dietary factors exert effects largely independent of genetic predisposition. These findings provide robust epidemiological evidence supporting the limitation of SSB intake as a modifiable dietary strategy for KSD prevention, regardless of an individual's genetic risk profile. Our study contributes important insights that may inform clinical dietary counseling and public health recommendations aimed at reducing the substantial burden of KSD.

## Data Availability

The original contributions presented in the study are included in the article/Supplementary material, further inquiries can be directed to the corresponding authors.
