# Supplementary material for: Sweetened beverage intake and risk of incident kidney stone: results from the UK Biobank
Source: Front Nutr. 2026 May 21;13:1844118. doi: 10.3389/fnut.2026.1844118 (PMC13233203; doi:10.3389/fnut.2026.1844118)
Supplement: Supplementary file 1 [file Supplementary_file_1.docx]

Table S1. Inclusion criteria for identifying kidney stone formers in the uk biobank population

| **ICD-10 Codes** | **ICD-10 Description** | **OPCS codes** | **OPCS Description** |
| --- | --- | --- | --- |
| **N20.0** | Calculus of kidney | M06.1 | Open removal of calculus from kidney |
| **N20.1** | Calculus of ureter | M09.1 | Endoscopic ultrasound fragmentation of calculus of kidney |
| **N20.2** | Calculus of kidney with calculus of ureter | M09.2 | Endoscopic electrohydraulic shock wave fragmentation of calculus of kidney |
| **N20.9** | Urinary calculus, unspecified | M09.3 | Endoscopic laser fragmentation of calculus of kidney |
| **N23** | Unspecified Renal Colic | M09.4 | Endoscopic extraction of calculus of kidney |
|  |  | M09.8 | Other specified therapeutic endoscopic operations on calculus of kidney |
|  |  | M09.9 | Unspecified therapeutic endoscopic operations on calculus of kidney |
| **Self-reported operation code** | **Self-reported Description** | M14.1 | Extracorporeal shock wave lithotripsy of calculus of kidney |
| **1197** | Percutaneous/open kidney stone surgery/lithotripsy | M14.8 | Other specified extracorporeal fragmentation of calculus of kidney |
|  |  | M14.9 | Unspecified extracorporeal fragmentation of calculus of kidney |
|  |  | M16.4 | Percutaneous nephrolithotomy |
|  |  | M27.1 | Ureteroscopic laser fragmentation of calculus of ureter |
|  |  | M27.2 | Ureteroscopic fragmentation of calculus of ureter |
|  |  | M27.3 | Ureteroscopic extraction of calculus of ureter |
|  |  | M28.1 | Endoscopic laser fragmentation of calculus of ureter |
|  |  | M28.2 | Endoscopic fragmentation of calculus of ureter |
|  |  | M28.3 | Endoscopic extraction of calculus of ureter |
|  |  | M28.4 | Endoscopic catheter drainage of calculus of ureter |
|  |  | M28.8 | Other specified other endoscopic removal of calculus from ureter |
|  |  | M28.9 | Unspecified other endoscopic removal of calculus from ureter |
|  |  | M31.1 | Extracorporeal shock wave lithotripsy of calculus of ureter |
|  |  | M31.8 | Other specified extracorporeal fragmentation of calculus of ureter |
|  |  | M31.9 | Unspecified extracorporeal fragmentation of calculus of ureter |
|  |  | M26.1 | Nephroscopic laser fragmentation of calculus of ureter |
|  |  | M26.2 | Nephroscopic fragmentation of calculus of ureter NEC |
|  |  | M26.3 | Nephroscopic extraction of calculus of ureter |
|  |  | M28.5 | Endoscopic drainage of calculus of ureter by dilation of ureter |
|  |  | M28.8 | Other specified other endoscopic removal of calculus from ureter |

Criteria were based on the reference: Liu Y, Ku PW, Li Z, Yang H, Zhang T, Chen L, Xia Y, Bai S. Intensity-Specific Physical Activity Measured by Accelerometer, Genetic Susceptibility, and the Risk of Kidney Stone Disease: Results from the UK Biobank. Am J Kidney Dis. 2024 Oct;84(4):437-446.e1.

Table S2. Information on genetic variants associated with kidney stone disease

|  | **ID** | **Position** | **Ref_Allele** | **Alt_Allele** | **Effect_Size** | **P_Value** |
| --- | --- | --- | --- | --- | --- | --- |
| **1** | rs10917002 | 21836340 | T | C | 0.053078443 | 3.45×10^-11^ |
| **2** | rs780093 | 27742603 | T | C | 0.041392685 | 1.31×10^-13^ |
| **2** | rs13003198 | 234257105 | T | C | 0.045322979 | 3.89×10^-11^ |
| **4** | rs1481012 | 89039082 | G | A | 0.045322979 | 2.79×10^-8^ |
| **5** | rs56235845 | 176798040 | G | T | 0.064457989 | 2.64×10^-21^ |
| **6** | rs1155347 | 39146230 | C | T | 0.053078443 | 8.54×10^-11^ |
| **6** | rs77648599 | 160624115 | G | T | 0.113943352 | 5.39×10^-10^ |
| **7** | rs12539707 | 27626165 | T | C | 0.049218023 | 1.09×10^-10^ |
| **7** | rs12666466 | 30916430 | G | C | 0.075546961 | 3.26×10^-8^ |
| **11** | rs4529910 | 111243102 | T | G | 0.037426498 | 4.25×10^-10^ |
| **13** | rs1037271 | 42779410 | C | T | 0.06069784 | 1.29×10^-24^ |
| **15** | rs578595 | 53997089 | C | A | 0.037426498 | 6.26×10^-11^ |
| **16** | rs77924615 | 20392332 | A | G | 0.056904851 | 1.14×10^-13^ |
| **16** | rs889299 | 23381914 | G | A | 0.037426498 | 1.55×10^-8^ |
| **17** | rs1010269 | 59448945 | G | A | 0.053078443 | 3.71×10^-15^ |
| **17** | rs4793434 | 70352537 | G | C | 0.037426498 | 4.52×10^-9^ |
| **19** | rs3760702 | 14588237 | A | G | 0.037426498 | 1.98×10^-9^ |
| **20** | rs17216707 | 52732362 | T | C | 0.075546961 | 7.82×10^-18^ |
| **21** | rs12626330 | 37835982 | G | C | 0.06069784 | 7.24×10^-21^ |
| **22** | rs13054904 | 23410918 | A | T | 0.056904851 | 4.49×10^-12^ |

The data source: Liu Y, Ku PW, Li Z, Yang H, Zhang T, Chen L, Xia Y, Bai S. Intensity-Specific Physical Activity Measured by Accelerometer, Genetic Susceptibility, and the Risk of Kidney Stone Disease: Results from the UK Biobank. Am J Kidney Dis. 2024 Oct;84(4):437-446.e1.

Table S3. Missing Data Summary and Imputation Strategies

| **Variable** | **Missing count** | **Missing rate** | **Variable type** | **Missing mechanism** | **Strategy** |
| --- | --- | --- | --- | --- | --- |
| **BMI** | 473 | 0.25% | Numeric | MCAR | Mean imputation |
| **Drinking_status** | 174 | 0.09% | Factor | MCAR | Mode imputation |
| **Ethnicity** | 623 | 0.32% | Factor | MCAR | Mode imputation |
| **Smoking_status** | 503 | 0.26% | Factor | MCAR | Mode imputation |
| **Tdi** | 241 | 0.13% | Numeric | MCAR | Mean imputation |
| **Household_income** | 19741 | 10.29% | Factor | MCAR | MICE |
| **Physical_activity** | 1800 | 0.94% | Factor | MCAR | Mode imputation |
| **Score_diet** | 71 | 0.04% | Numeric | MCAR | Mean imputation |
| **eGFR** | 9182 | 4.79% | Numeric | MCAR | MICE |
| **Drinking_water** | 66 | 0.03% | Factor | MCAR | Mode imputation |
| **Other variables** | 0 | 0.00% | Numeric/Factor | Complete | No action needed |

Table S4. Baseline characteristics of participants according to intake of artificially sweetened beverage and natural juice intake (n =191,863)

|  | **Intake categories of artificially-sweetened beverage** | | | | **Intake categories of naturally sweet juices** | | | |
| --- | --- | --- | --- | --- | --- | --- | --- | --- |
|  | **None** | **>0-1 units/d** | **>1-2 units/d** | **>2 units/d** | **None** | **>0-1 units/d** | **>1-2 units/d** | **>2 units/d** |
| **n** | 152166 | 29271 | 6915 | 3511 | 92823 | 85251 | 11852 | 1937 |
| **Age (mean (sd))** | 56.36 (7.87) | 54.67 (7.95) | 53.44 (8.04) | 52.44 (7.93) | 55.64 (7.98) | 56.29 (7.88) | 55.64 (8.00) | 55.21 (8.04) |
| **Bmi (mean (sd))** | 26.91 (4.62) | 26.52 (4.38) | 27.98 (4.90) | 29.18 (5.43) | 30.36 (6.14) | 27.22 (4.79) | 26.61 (4.43) | 26.61 (4.38) |
| **Underweight** | 928 (0.6) | 85 (0.3) | 10 (0.1) | 7 (0.2) | 520 (0.6) | 441 (0.5) | 58 (0.5) | 11 (0.6) |
| **Normal** | 60676 (39.9) | 8246 (28.2) | 1491 (21.6) | 647 (18.4) | 32197 (34.7) | 33546 (39.3) | 4634 (39.1) | 683 (35.3) |
| **Overweight** | 63106 (41.5) | 12722 (43.5) | 2870 (41.5) | 1309 (37.3) | 38679 (41.7) | 35518 (41.7) | 4984 (42.1) | 826 (42.6) |
| **Obese** | 27456 (18.0) | 8218 (28.1) | 2544 (36.8) | 1548 (44.1) | 21427 (23.1) | 15746 (18.5) | 2176 (18.4) | 417 (21.5) |
| **Gender (%)** |  |  |  |  |  |  |  |  |
| **Female** | 82661 (54.3) | 17128 (58.5) | 4057 (58.7) | 2093 (59.6) | 53420 (57.6) | 45854 (53.8) | 5826 (49.2) | 839 (43.3) |
| **Male** | 69505 (45.7) | 12143 (41.5) | 2858 (41.3) | 1418 (40.4) | 39403 (42.4) | 39397 (46.2) | 6026 (50.8) | 1098 (56.7) |
| **Drinking status (%)** |  |  |  |  |  |  |  |  |
| **Never** | 4733 (3.1) | 922 (3.1) | 239 (3.5) | 183 (5.2) | 3208 (3.5) | 2338 (2.7) | 410 (3.5) | 121 (6.2) |
| **Previous** | 4318 (2.8) | 879 (3.0) | 316 (4.6) | 224 (6.4) | 3215 (3.5) | 2052 (2.4) | 368 (3.1) | 102 (5.3) |
| **Current** | 143115 (94.1) | 27470 (93.8) | 6360 (92.0) | 3104 (88.4) | 86400 (93.1) | 80861 (94.9) | 11074 (93.4) | 1714 (88.5) |
| **Ethnicity (%)** |  |  |  |  |  |  |  |  |
| **Non_white** | 6467 (4.2) | 1169 (4.0) | 236 (3.4) | 132 (3.8) | 4219 (4.5) | 2942 (3.5) | 605 (5.1) | 238 (12.3) |
| **White** | 145699 (95.8) | 28102 (96.0) | 6679 (96.6) | 3379 (96.2) | 88604 (95.5) | 82309 (96.5) | 11247 (94.9) | 1699 (87.7) |
| **Tdi (mean (sd))** | -1.59 (2.86) | -1.64 (2.84) | -1.52 (2.90) | -1.18 (3.06) | -1.44 (2.93) | -1.77 (2.77) | -1.53 (2.92) | -0.86 (3.26) |
| **Smoking status (%)** |  |  |  |  |  |  |  |  |
| **Never** | 87076 (57.2) | 16613 (56.8) | 3771 (54.5) | 1858 (52.9) | 50235 (54.1) | 50649 (59.4) | 7242 (61.1) | 1192 (61.5) |
| **Previous** | 53160 (34.9) | 10525 (36.0) | 2566 (37.1) | 1290 (36.7) | 33925 (36.5) | 29259 (34.3) | 3763 (31.7) | 594 (30.7) |
| **Current** | 11930 (7.8) | 2133 (7.3) | 578 (8.4) | 363 (10.3) | 8663 (9.3) | 5343 (6.3) | 847 (7.1) | 151 (7.8) |
| **Education (%)** |  |  |  |  |  |  |  |  |
| **College** | 66714 (43.8) | 11637 (39.8) | 2557 (37.0) | 1272 (36.2) | 34164 (36.8) | 40542 (47.6) | 6435 (54.3) | 1039 (53.6) |
| **Other levels** | 71982 (47.3) | 15122 (51.7) | 3766 (54.5) | 1917 (54.6) | 48413 (52.2) | 38842 (45.6) | 4762 (40.2) | 770 (39.8) |
| **Unknown** | 13470 (8.9) | 2512 (8.6) | 592 (8.6) | 322 (9.2) | 10246 (11.0) | 5867 (6.9) | 655 (5.5) | 128 (6.6) |
| **Household income， €/y (%)** |  |  |  |  |  |  |  |  |
| **Less than 18,000** | 23609 (15.5) | 4157 (14.2) | 979 (14.2) | 565 (16.1) | 16050 (17.3) | 11414 (13.4) | 1549 (13.1) | 297 (15.3) |
| **18,000 to 30,999** | 37020 (24.3) | 6783 (23.2) | 1577 (22.8) | 827 (23.6) | 23120 (24.9) | 20079 (23.6) | 2553 (21.5) | 455 (23.5) |
| **31,000 to 51,999** | 43430 (28.5) | 8320 (28.4) | 1994 (28.8) | 1024 (29.2) | 26274 (28.3) | 24724 (29.0) | 3240 (27.3) | 530 (27.4) |
| **52,000 to 100,000** | 37113 (24.4) | 7671 (26.2) | 1835 (26.5) | 865 (24.6) | 21476 (23.1) | 22189 (26.0) | 3352 (28.3) | 467 (24.1) |
| **Greater than 100,000** | 10994 (7.2) | 2340 (8.0) | 530 (7.7) | 230 (6.6) | 5903 (6.4) | 6845 (8.0) | 1158 (9.8) | 188 (9.7) |
| **Physical activity (%)** |  |  |  |  |  |  |  |  |
| **Non** | 57118 (37.5) | 11431 (39.1) | 2755 (39.8) | 1479 (42.1) | 35886 (38.7) | 32049 (37.6) | 4196 (35.4) | 652 (33.7) |
| **Yes** | 95048 (62.5) | 17840 (60.9) | 4160 (60.2) | 2032 (57.9) | 56937 (61.3) | 53202 (62.4) | 7656 (64.6) | 1285 (66.3) |
| **Score diet (mean (sd))** | 2.90 (1.28) | 2.80 (1.25) | 2.72 (1.25) | 2.65 (1.28) | 2.81 (1.29) | 2.91 (1.26) | 3.04 (1.29) | 3.16 (1.30) |
| **Egfr (mean (sd))** | 95.60 (12.42) | 96.29 (12.82) | 97.19 (12.94) | 98.52 (13.34) | 95.75 (12.68) | 95.78 (12.35) | 96.54 (12.50) | 96.61 (12.70) |
| **Drinking water (%)** |  |  |  |  |  |  |  |  |
| **<3** | 73400 (48.2) | 14416 (49.3) | 3141 (45.4) | 1437 (40.9) | 45270 (48.8) | 40977 (48.1) | 5354 (45.2) | 793 (40.9) |
| **>=3** | 66641 (43.8) | 12716 (43.4) | 3323 (48.1) | 1850 (52.7) | 39975 (43.1) | 37812 (44.4) | 5709 (48.2) | 1034 (53.4) |
| **Unknown** | 12125 (8.0) | 2139 (7.3) | 451 (6.5) | 224 (6.4) | 7578 (8.2) | 6462 (7.6) | 789 (6.7) | 110 (5.7) |

Abbreviations: BMI; body mass index; eGFR, estimated glomerular filtration rate; LDL-C, low-density lipoprotein cholesterol; TDI, Townsend Deprivation Index.

1: Body weight status was defined by BMI (≤20 kg/m2 to be underweight, >20 and≤25 to be normal weight, >25 and ≤30 to be overweight, and ＞30 to be obesity).

Table S5. Risk of incident kidney stone by category of beverage intake stratified by age, gender, drinking water, eGFR, and BMI

| **Subgroup** | **N** | **SSB** | | **ASB** | | **NJ** | |
| --- | --- | --- | --- | --- | --- | --- | --- |
|  |  | **HR (95% CI)** | **P-interaction** | **HR (95% CI)** | **P-interaction** | **HR (95% CI)** | **P-interaction** |
| Age |  |  | 0.971 |  | 0.542 |  | 0.002 |
| <60 years | 115574 |  |  |  |  |  |  |
| 0 |  | (Reference) |  | (Reference) |  | (Reference) |  |
| >0~1 |  | 1.17 (1.03, 1.33) |  | 1.07 (0.92, 1.24) |  | 0.87 (0.76, 1.01) |  |
| >1~2 |  | 1.13 (0.87, 1.46) |  | 1.44 (0.90, 2.31) |  | 0.66 (0.47, 0.93) |  |
| >2 |  | 0.92 (0.64, 1.32) |  | 1.01 (0.50, 2.05) |  | 1.25 (0.69, 2.28) |  |
| >=60 years | 76289 |  |  |  |  |  |  |
| 0 |  | (Reference) |  | (Reference) |  | (Reference) |  |
| >0~1 |  | 1.19 (1.02, 1.39) |  | 1.02 (0.84, 1.25) |  | 0.87 (0.76, 0.99) |  |
| >1~2 |  | 1.50 (1.11, 2.02) |  | 1.55 (1.12, 2.16) |  | 0.99 (0.80, 1.22) |  |
| >2 |  | 1.68 (1.06, 2.66) |  | 0.99 (0.54, 1.80) |  | 0.95 (0.64, 1.41) |  |
| Gender |  |  | 0.602 |  | 0.226 |  | 0.861 |
| Female | 105939 |  |  |  |  |  |  |
| 0 |  | (Reference) |  | (Reference) |  | (Reference) |  |
| >0~1 |  | 1.26 (1.07, 1.48) |  | 1.19 (0.99, 1.43) |  | 0.98 (0.85, 1.14) |  |
| >1~2 |  | 1.64 (1.21, 2.22) |  | 1.16 (0.83, 1.63) |  | 1.01 (0.73, 1.41) |  |
| >2 |  | 1.83 (1.16, 2.87) |  | 0.75 (0.44, 1.29) |  | 1.15 (0.55, 2.44) |  |
| Male | 885924 |  |  |  |  |  |  |
| 0 |  | (Reference) |  | (Reference) |  | (Reference) |  |
| >0~1 |  | 1.13 (1.00, 1.28) |  | 0.97 (0.83, 1.14) |  | 1.02 (0.91, 1.15) |  |
| >1~2 |  | 1.44 (1.17, 1.78) |  | 1.32 (1.02, 1.71) |  | 1.01 (0.81, 1.28) |  |
| >2 |  | 1.39 (1.02, 1.90) |  | 1.07 (0.73, 1.71) |  | 1.43 (0.93, 2.19) |  |
| Drinking water |  |  | 0.232 |  | 0.956 |  | 0.680 |
| <3L | 92394 |  |  |  |  |  |  |
| 0 |  | (Reference) |  | (Reference) |  | (Reference) |  |
| >0~1 |  | 1.15 (1.00, 1.32) |  | 0.99 (0.84, 2.00) |  | 1.05 (0.92, 1.20) |  |
| >1~2 |  | 1.62 (1.27, 2.05) |  | 1.24 (0.92, 1.67) |  | 1.01 (0.76, 1.33) |  |
| >2 |  | 1.39 (0.93, 2.08) |  | 1.04 (0.66, 1.63) |  | 1.23 (0.67, 2.23) |  |
| >=3L | 84530 |  |  |  |  |  |  |
| 0 |  | (Reference) |  | (Reference) |  | (Reference) |  |
| >0~1 |  | 1.23 (1.05, 1.43) |  | 1.07 (0.89, 1.28) |  | 0.99 (0.86, 1.14) |  |
| >1~2 |  | 1.63 (1.26, 2.11) |  | 1.20 (0.89, 1.63) |  | 0.98 (0.74, 1.31) |  |
| >2 |  | 1.66 (1.15, 2.39) |  | 0.84 (0.54, 1.32) |  | 1.53 (0.93, 2.53) |  |
| Unkown | 14938 |  |  |  |  |  |  |
| 0 |  | (Reference) |  | (Reference) |  | (Reference) |  |
| >0~1 |  | 1.27 (0.90, 1.80) |  | 1.18 (0.76, 1.82) |  | 1.09 (0.78, 1.53) |  |
| >1~2 |  | 0.63 (0.23, 1.72) |  | 1.47 (0.68, 3.18) |  | 1.92 (1.06, 3.43) |  |
| >2 |  | 2.91 (1.33, 6.35) |  | 0.82 (0.20, 3.36) |  | 2.05 (0.50, 8.42) |  |
| eGFR^a^ |  |  | <0.001 |  | 0.732 |  | 0.723 |
| <98.1 ml/min/1.73m^2^ | 95718 |  |  |  |  |  |  |
| 0 |  | (Reference) |  | (Reference) |  | (Reference) |  |
| >0~1 |  | 1.09 (0.95, 1.26) |  | 1.01 (0.85, 1.20) |  | 1.00 (0.88, 1.14) |  |
| >1~2 |  | 1.47 (1.14, 1.89) |  | 1.22 (0.90, 1.67) |  | 1.05 (0.90, 1.37) |  |
| >2 |  | 1.19 (0.78, 1.83) |  | 1.10 (0.70, 1.74) |  | 1.98 (0.59, 1.96) |  |
| >=98.1 ml/min/1.73m^2^ | 96145 |  |  |  |  |  |  |
| 0 |  | (Reference) |  | (Reference) |  | (Reference) |  |
| >0~1 |  | 1.27 (1.10, 1.46) |  | 1.09 (0.92, 1.29) |  | 1.02 (0.89, 1.16) |  |
| >1~2 |  | 1.55 (1.22, 1.97) |  | 1.28 (0.98, 1.68) |  | 0.98 (0.75, 1.28) |  |
| >2 |  | 1.79 (1.30, 2.47) |  | 0.84 (0.55, 1.28) |  | 1.58 (0.98, 2.53) |  |
| BMI |  |  | 0.673 |  | 0.022 |  | 0.270 |
| <25.0 kg/m^2^ | 72090 |  |  |  |  |  |  |
| >0~1 |  | 1.28 (1.05, 1.56) |  | 1.26 (0.98, 1.63) |  | 1.04 (0.87, 1.25) |  |
| >1~2 |  | 1.67 (1.16, 2.41) |  | 0.61 (0.27, 1.36) |  | 1.01 (0.69, 1.46) |  |
| >2 |  | 2.04 (1.19, 3.50) |  | 0.47 (0.12, 1.90) |  | 0.59 (0.18, 1.85) |  |
| >=25.0 kg/m^2^ | 119773 |  |  |  |  |  |  |
| >0~1 |  | 1.14 (1.02, 1.28) |  | 1.05 (0.92, 1.21) |  | 0.98 (0.89, 1.10) |  |
| >1~2 |  | 1.49 (1.26, 1.81) |  | 1.48 (1.19, 1.82) |  | 1.00 (0.81, 1.25) |  |
| >2 |  | 1.47 (1.10, 1.97) |  | 1.14 (0.83, 1.57) |  | 1.58 (1.06, 2.34) |  |

Adjusted for age, gender, BMI, ethnicity, education, drinking status, Townsend deprivation index, household income, smoking status, physical activity, healthy diet score and eGFR

^a^eGFR median value wield 98.1 ml/min/1.73m^2^.

Table S6. Risk of incident kidney stone by category of beverage intake excluded for missing any covariate data

|  | **Cases/total** | **Person-years** | **Model** |
| --- | --- | --- | --- |
| **SSB (units/d)** |  |  |  |
| **0, HR (95% CI)** | 1018/109164 | 1482238 | 1(reference) |
| **>0-1, HR (95% CI)** | 473/41860 | 568334 | 1.17(1.05, 1.30) |
| **>1-2, HR (95% CI)** | 119/7560 | 102119 | 1.50(1.24, 1.81) |
| **>2, HR (95% CI)** | 55/2950 | 2950 | 1.63(1.24, 2.15) |
| **P-trend** |  |  | <0.001 |
| **ASB (units/d)** |  |  |  |
| **0, HR (95% CI)** | 1275/127925 | 1735587 | 1(reference) |
| **>0-1, HR (95% CI)** | 276/24790 | 336939 | 1.07(0.94, 1.22) |
| **>1-2, HR (95% CI)** | 75/5832 | 79065 | 1.16(0.91, 1.47) |
| **>2, HR (95% CI)** | 39/2987 | 40518 | 1.07(0.77, 1.48) |
| **P-trend** |  |  | 0.180 |
| **NJ (units/d)** |  |  |  |
| **0, HR (95% CI)** | 811/77404 | 1047216 | 1(reference) |
| **>0-1, HR (95% CI)** | 727/723383 | 985336 | 1.01(0.91, 1.12) |
| **>1-2, HR (95% CI)** | 102/10119 | 137615 | 1.00(0.81, 1.23) |
| **>2, HR (95% CI)** | 25/1628 | 21943 | 1.41(0.95, 2.11) |
| **P-trend** |  |  | 0.446 |

Abbreviations: HR, hazard ratio; NA, not applicable.

Model: adjusted for age, gender, BMI, ethnicity, education, drinking status, Townsend deprivation index, household income, smoking status, physical activity, healthy diet score and eGFR.

Table S7. Risk of incident kidney stone by category of beverage intake among the participants who conducted two or more dietary assessments

|  | **Cases/total** | **Person-years** | **Model** |
| --- | --- | --- | --- |
| **SSB (units/d)** |  |  |  |
| **0, HR (95% CI)** | 629/70638 | 1482238 | 1(reference) |
| **>0-1, HR (95% CI)** | 433/38708 | 525867 | 1.21(1.06, 1.37) |
| **>1-2, HR (95% CI)** | 97/5559 | 102119 | 1.71(1.38, 2.13) |
| **>2, HR (95% CI)** | 25/1694 | 22661 | 1.36(0.91, 2.04) |
| **P-trend, HR (95% CI)** |  |  | <0.001 |
| **ASB (units/d)** |  |  |  |
| **0, HR (95% CI)** | 845/87965 | 1198601 | 1(reference) |
| **>0-1, HR (95% CI)** | 256/303848 | 22343 | 1.14(0.98, 1.31) |
| **>1-2, HR (95% CI)** | 62/4387 | 79065 | 1.32(1.01, 1.71) |
| **>2, HR (95% CI)** | 21/1904 | 26023 | 0.92(0.60, 1.43) |
| **P-trend** |  |  | 0.084 |
| **NJ (units/d)** |  |  |  |
| **0, HR (95% CI)** | 477/46887 | 638000 | 1(reference) |
| **>0-1, HR (95% CI)** | 602/60301 | 821923 | 1.02(0.90, 1.15) |
| **>1-2, HR (95% CI)** | 92/8399 | 114397 | 1.09(0.87, 1.37) |
| **>2, HR (95% CI)** | 13/1012 | 13720 | 1.21(0.70, 2.10) |
| **P-trend** |  |  | 0.400 |

Abbreviations: HR, hazard ratio; NA, not applicable.

Model: adjusted for age, gender, BMI, ethnicity, education, drinking status, Townsend deprivation index, household income, smoking status, physical activity, healthy diet score and eGFR.

Table S8. Risk of incident kidney stones by beverage intake category mutually adjusted for three types of sugary beverages

|  | **Cases/total** | **Person-years** | **Model** |
| --- | --- | --- | --- |
| **SSB (units/d)** |  |  |  |
| **0, HR (95% CI)** | 1236/129675 | 1756946 | 1(reference) |
| **>0-1, HR (95% CI)** | 578/49635 | 672393 | 1.17(1.06, 1.29) |
| **>1-2, HR (95% CI)** | 147/9013 | 121448 | 1.50(1.26, 1.78) |
| **>2, HR (95% CI)** | 63/3540 | 47198 | 1.50(1.16, 1.94) |
| **P-trend** |  |  | <0.001 |
| **ASB (units/d)** |  |  |  |
| **0, HR (95% CI)** | 1552/152166 | 2060048 | 1(reference) |
| **>0-1, HR (95% CI)** | 330/29271 | 397081 | 1.03(0.91, 1.16) |
| **>1-2, HR (95% CI)** | 100/6915 | 93341 | 1.23(1.00, 1.51) |
| **>2, HR (95% CI)** | 42/3511 | 47515 | 0.94(0.69, 1.28) |
| **P-trend** |  |  | 0.519 |
| **NJ (units/d)** |  |  |  |
| **0, HR (95% CI)** | 997/92823 | 1252821 | 1(reference) |
| **>0-1, HR (95% CI)** | 875/85251 | 1158284 | 1.00(0.91, 1.10) |
| **>1-2, HR (95% CI)** | 123/11852 | 160837 | 1.00(0.82, 1.20) |
| **>2, HR (95% CI)** | 29/1931 | 26044 | 1.33(0.92, 1.92) |
| **P-trend** |  |  | 0.137 |

Abbreviations: HR, hazard ratio; NA, not applicable.

Model: adjusted for age, gender, BMI, ethnicity, education, drinking status, Townsend deprivation index, household income, smoking status, physical activity, healthy diet score and eGFR.

Table S9. Risk of incident kidney stone by category of beverage intake using Fine-Gray competing risks model.

|  | **Cases/total** | **Person-years** | **Model** |
| --- | --- | --- | --- |
| **SSB (units/d)** |  |  |  |
| **0, HR (95% CI)** | 1236/129675 | 1756946 | 1(reference) |
| **>0-1, HR (95% CI)** | 578/49635 | 672393 | 1.17(1.06, 1.30) |
| **>1-2, HR (95% CI)** | 147/9013 | 121448 | 1.51(1.27, 1.79) |
| **>2, HR (95% CI)** | 63/3540 | 47198 | 1.50(1.16, 1.93) |
| **P-trend** |  |  | <0.001 |
| **ASB (units/d)** |  |  |  |
| **0, HR (95% CI)** | 1552/152166 | 2060048 | 1(reference) |
| **>0-1, HR (95% CI)** | 330/29271 | 397081 | 1.06(0.94, 1.19) |
| **>1-2, HR (95% CI)** | 100/6915 | 93341 | 1.25(1.02, 1.54) |
| **>2, HR (95% CI)** | 42/3511 | 47515 | 0.94(0.69, 1.28) |
| **P-trend** |  |  | 0.198 |
| **NJ (units/d)** |  |  |  |
| **0, HR (95% CI)** | 997/92823 | 1252821 | 1(reference) |
| **>0-1, HR (95% CI)** | 875/85251 | 1158284 | 1.02(0.93, 1.12) |
| **>1-2, HR (95% CI)** | 123/11852 | 160837 | 1.02(0.84, 1.23) |
| **>2, HR (95% CI)** | 29/1931 | 26044 | 1.34(0.93, 1.94) |
| **P-trend** |  |  | 0.363 |

Abbreviations: HR, hazard ratio; NA, not applicable.

Model: adjusted for age, gender, BMI, ethnicity, education, drinking status, Townsend deprivation index, household income, smoking status, physical activity, healthy diet score and eGFR.

Figure S1. Association of PRS and the risk of incident kidney stone

(n =191,863)


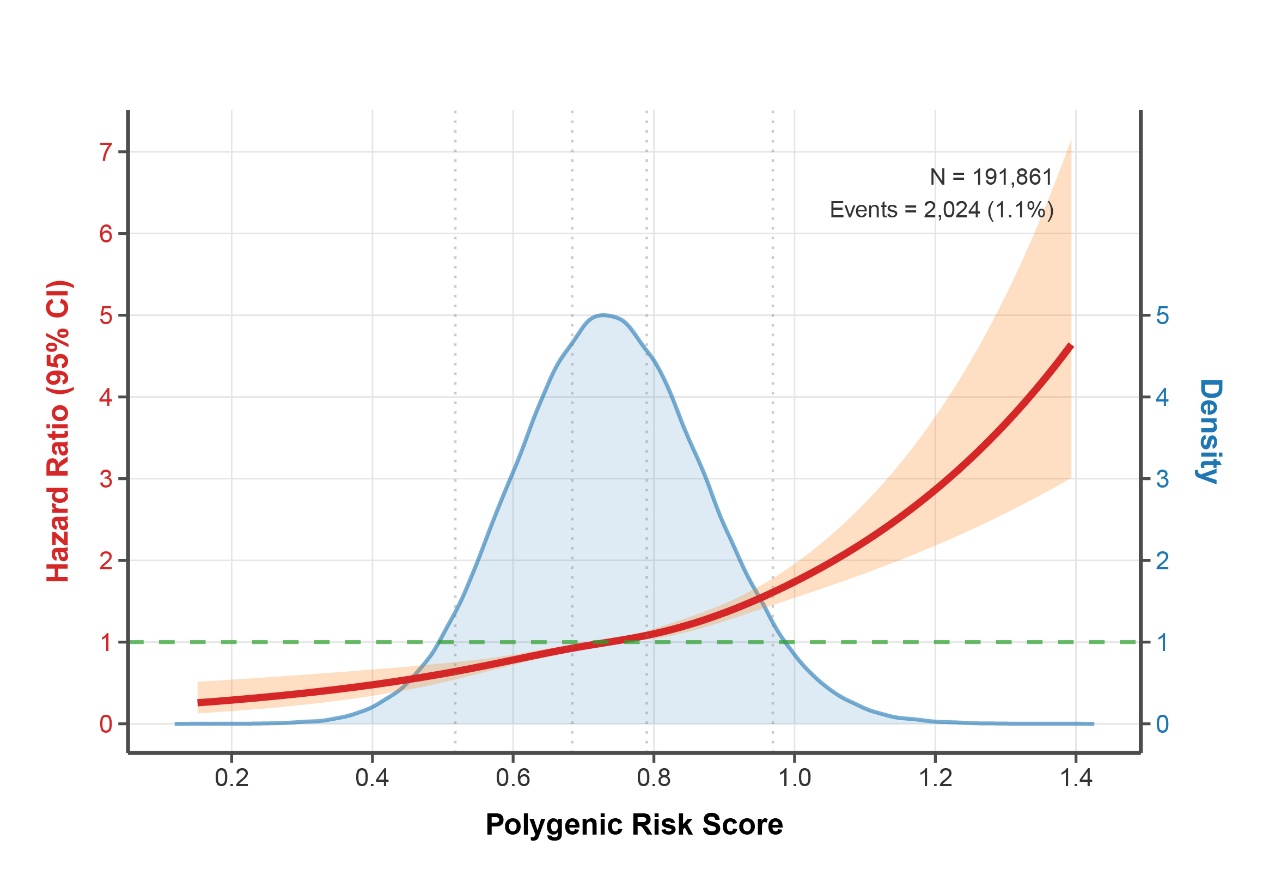


Abbreviations: PRS, polygenic risk score; HR, hazard ratio; CI, confidence interval.

Adjusted for age, gender, and the first 10 principal components of ancestry.

Fig S2 Joint effects of polygenic risk score (PRS) with artificially-sweetened beverage intake on kidney stone risk (n=191,863)


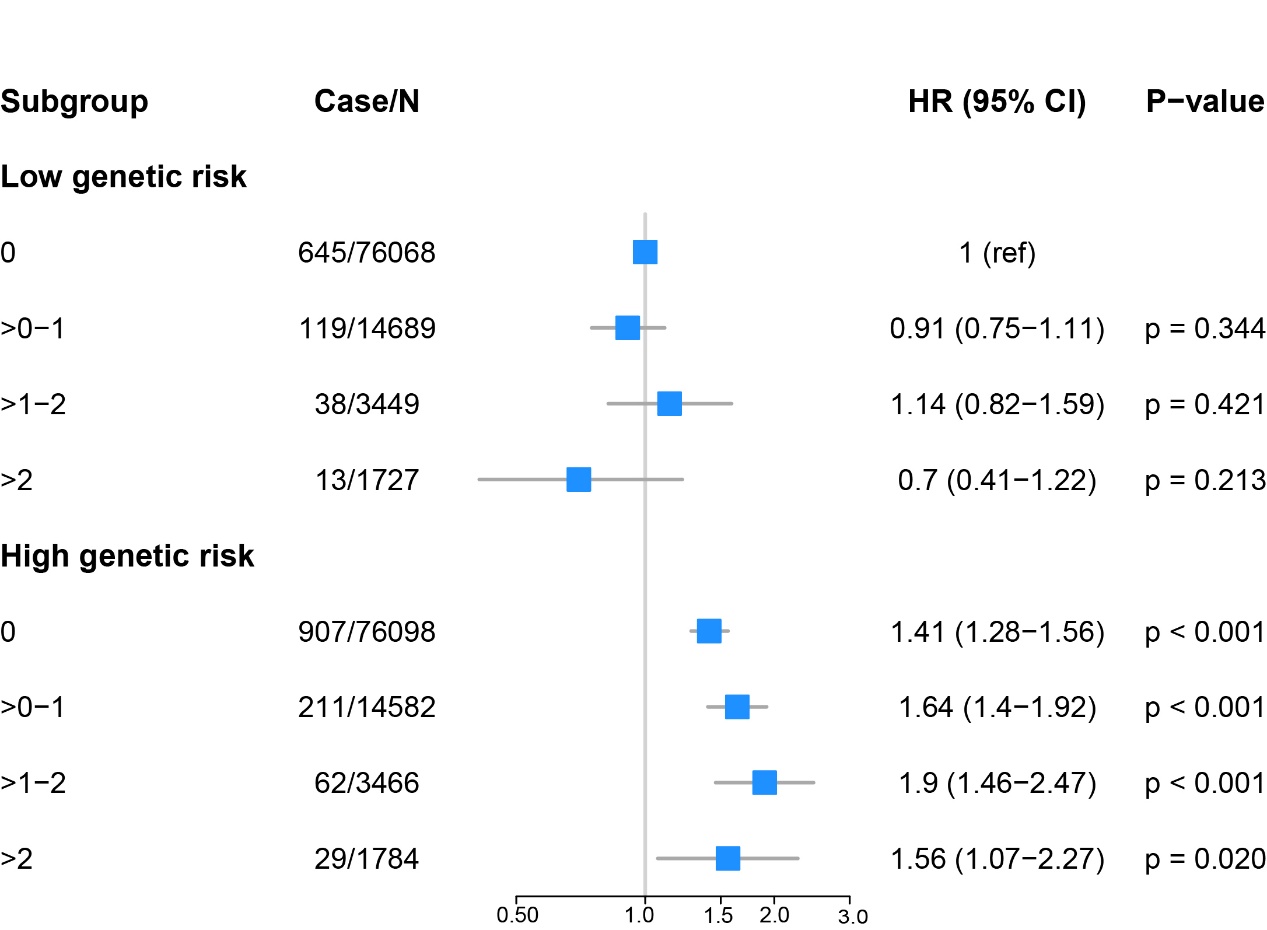


P-interactions = 0.133 for ASB ×PRS

The blue boxes indicate the HRs, and the gray whiskers represent the CIs. HRs were calculated from Cox proportional hazard models adjusted for age, gender, BMI, ethnicity, education, drinking status, Townsend deprivation index, household income, smoking status, physical activity, healthy diet score and eGFR

Fig S3. Joint effects of polygenic risk score (PRS) with naturally sweet juices intake on kidney stone risk (n=191,863)


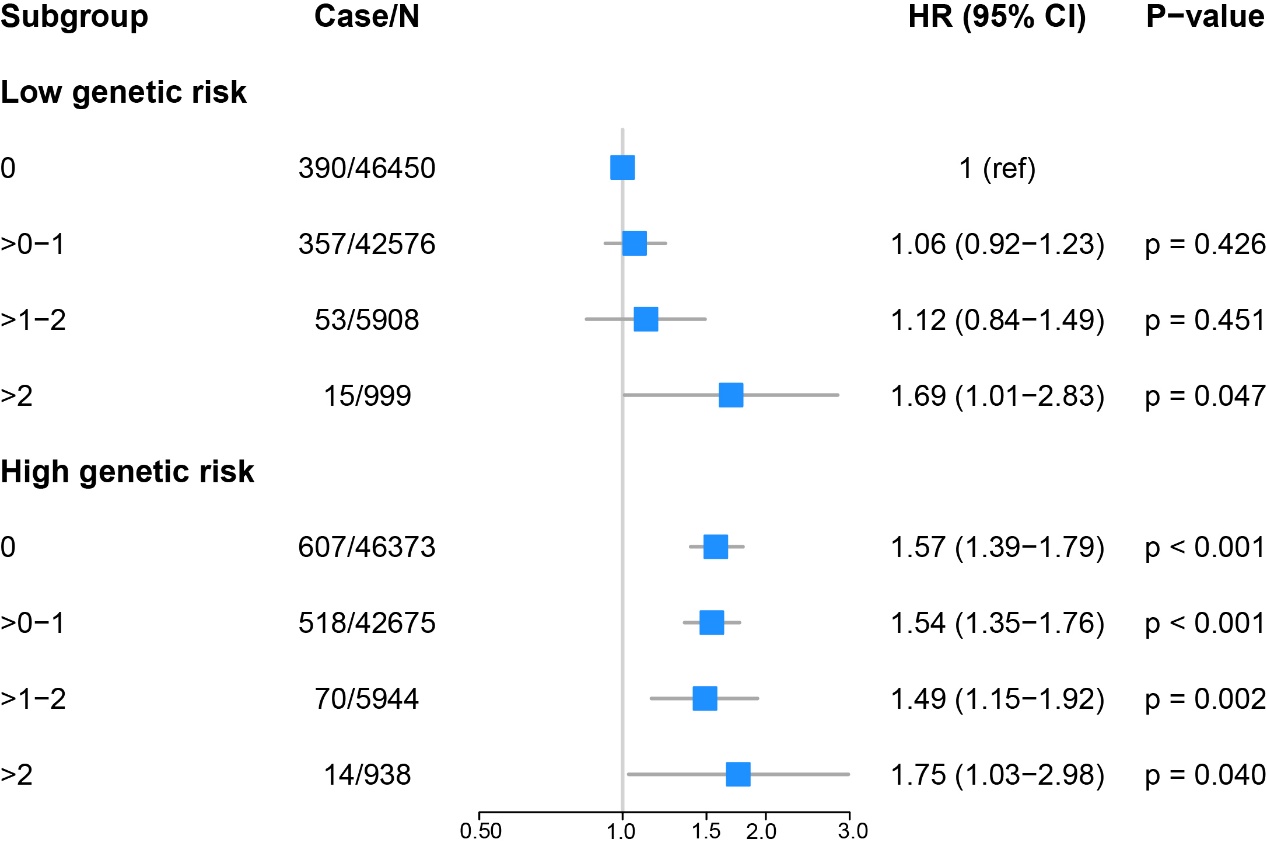


P-interactions = 0.537 for NJ ×PRS

The blue boxes indicate the HRs, and the gray whiskers represent the CIs. HRs were calculated from Cox proportional hazard models adjusted for age, gender, BMI, ethnicity, education, drinking status, Townsend deprivation index, household income, smoking status, physical activity, healthy diet score and eGFR
